# COVID-19 and Cancer Comorbidity: Therapeutic Opportunities and Challenges

**DOI:** 10.7150/thno.51471

**Published:** 2021-01-01

**Authors:** Anup S. Pathania, Philip Prathipati, Bakrudeen AA. Abdul, Srinivas Chava, Santharam S. Katta, Subash C. Gupta, Pandu R. Gangula, Manoj K. Pandey, Donald L. Durden, Siddappa N. Byrareddy, Kishore B. Challagundla

**Affiliations:** 1 Department of Biochemistry and Molecular Biology & The Fred and Pamela Buffett Cancer Center; University of Nebraska Medical Center, Omaha, NE 68198, USA.; 2 Laboratory of Bioinformatics, National Institutes of Biomedical Innovation, Health and Nutrition, Saito-Asagi Ibaraki City, Osaka 567-0085, Japan.; 3 Department of Biochemistry, Center for Research & Development, PRIST Deemed University, Vallam, Tamil Nadu 613403, India.; 4 Department of Biotechnology, School of Applied Sciences, REVA University, Rukmini Knowledge Park Kattigenahalli, Yelahanka, Bangalore, Karnataka 560064, India.; 5 Department of Biochemistry, Institute of Science, Banaras Hindu University, Varanasi, Uttar Pradesh 221005, India.; 6 Department of Oral Diagnostic Sciences and Research, School of Dentistry, Meharry Medical College, Nashville, TN 37208, USA.; 7 Department of Biomedical Sciences, Cooper Medical School of Rowan University, Camden, NJ 08103, USA.; 8 Levine Cancer Institute, Atrium Health, Charlotte, NC 28202, USA.; 9 Department of Pediatrics, University of California, San Diego, San Diego, CA 92093, USA.; 10 SignalRx Pharmaceuticals, Omaha, NE 68124, USA.; 11 Department of Pharmacology and Experimental Neuroscience, University of Nebraska Medical Center, Omaha, NE 68198, USA.; 12 The Children's Health Research Institute, University of Nebraska Medical Center, Omaha, NE, 68198, USA.

**Keywords:** COVID-19, coronaviruses, SARS-CoV-2, cancer, inflammation, comorbidity

## Abstract

The coronavirus disease 2019 (COVID-19) is a viral disease caused by a novel severe acute respiratory syndrome coronavirus 2 (SARS-CoV-2) that affects the respiratory system of infected individuals. COVID-19 spreads between humans through respiratory droplets produced when an infected person coughs or sneezes. The COVID-19 outbreak originated in Wuhan, China at the end of 2019. As of 29 Sept 2020, over 235 countries, areas or territories across the globe reported a total of 33,441,919 confirmed cases, and 1,003,497 confirmed deaths due to COVID-19. Individuals of all ages are at risk for infection, but in most cases disease severity is associated with age and pre-existing diseases that compromise immunity, like cancer. Numerous reports suggest that people with cancer can be at higher risk of severe illness and related deaths from COVID-19. Therefore, managing cancer care under this pandemic is challenging and requires a collaborative multidisciplinary approach for optimal care of cancer patients in hospital settings. In this comprehensive review, we discuss the impact of the COVID-19 pandemic on cancer patients, their care, and treatment. Further, this review covers the SARS-CoV-2 pandemic, genome characterization, COVID-19 pathophysiology, and associated signaling pathways in cancer, and the choice of anticancer agents as repurposed drugs for treating COVID-19.

## 1. COVID-19 Pandemic

**Figure 1:**
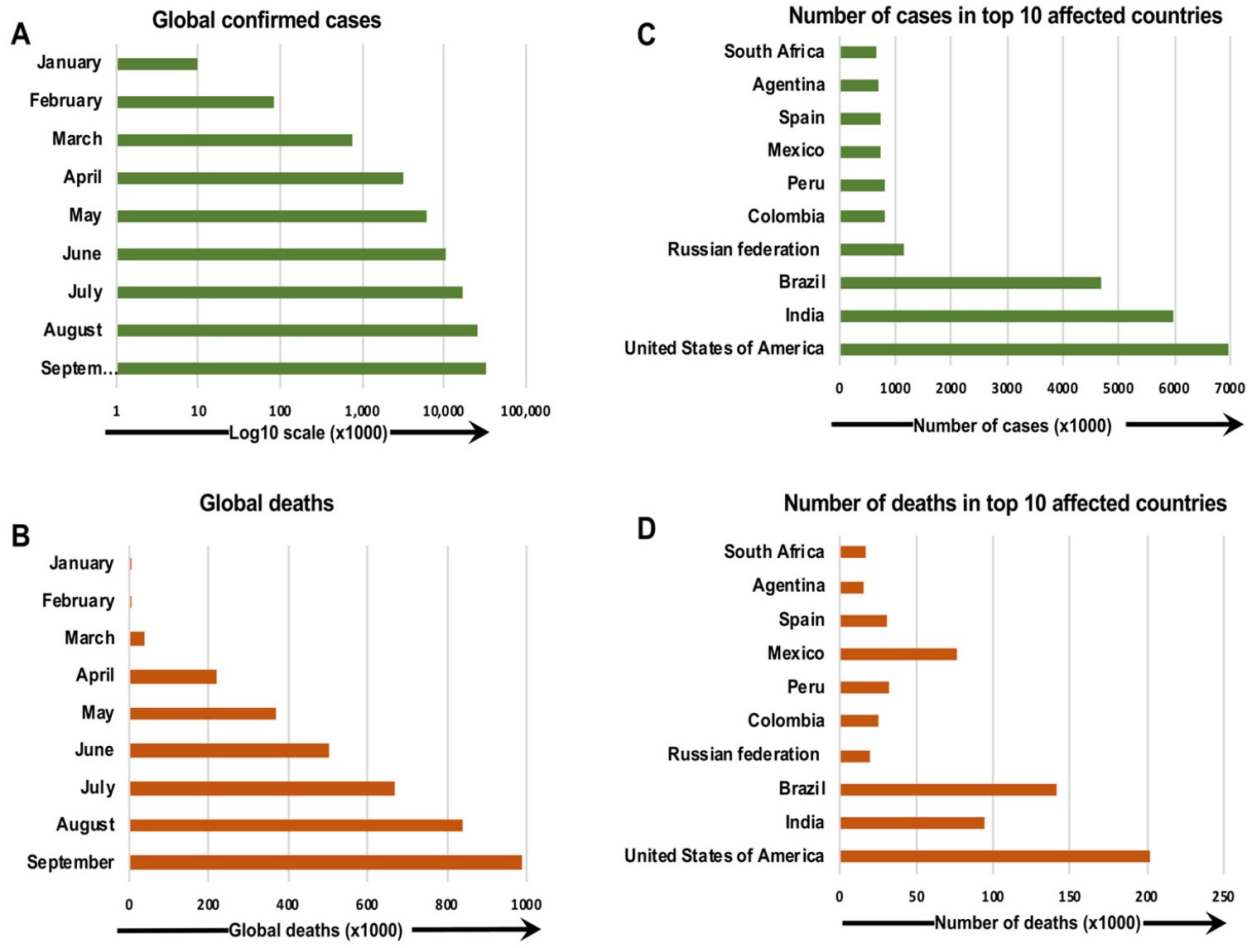
The confirmed COVID-19 cases and deaths around the world as of 30 September 2020. The number of confirmed COVID-19 cases and deaths per month (a, b), and countries (c, d), around the globe, are presented 185, 186.

The first outbreak causing the coronavirus disease 2019 (COVID-19) pandemic appeared in Wuhan, China in 2019. In December 2019, COVID-19 was identified in a group of people with pneumonia from the Huanan Seafood Wholesale Market in Wuhan, a city in the Hubei Province of China 1. In the United States (U.S.), the first confirmed case of severe acute respiratory syndrome coronavirus 2 (SARS-CoV-2) infection was reported on January 20, 2020 in a 35-year-old man who had traveled from Wuhan, China 2. Quickly, an increasing number of positive cases was reported in many other countries, and COVID-19 became a severe health emergency worldwide (Figure 1) 3-5. In March 2020, the World Health Organization (WHO) characterized COVID-19 as a pandemic and declared the outbreak a public health emergency of international concern 6.

The virus mainly spreads between people via nose or mouth secretions, including respiratory droplets or saliva, when an infected person coughs, sneezes, talks, or sings 7-9. Infection can also occur by touching a surface that has the virus on it and then touching one's own mouth, nose, or possibly eyes 10. Recent studies demonstrated that numerous bat coronaviruses (CoVs) infect humans without an additional carrier. SARS-CoV and Middle East respiratory syndrome coronavirus (MERS-CoV), which also originated in bats, were the most significant transmission outbreaks until SARS-CoV-2 outbreak in humans. The symptoms seen in COVID-19 patients are severe respiratory illness, fever, cough, shortness of breath, sore throat, congestion, fatigue, body aches, loss of taste or smell, and, in some, gastrointestinal distress 11, 12. Some patients exhibit pneumonia in both lungs, multi-organ failure, and even death 13-16. Individuals who have severe health conditions, like cancer, cardiovascular diseases, diabetes, and pulmonary diseases, are at higher risk of SARS-CoV-2 infection 17-21. At present, there is neither a vaccine nor specific antiviral treatments approved for COVID-19, but some medications have been authorized for emergency use in certain patients. This includes corticosteroids like dexamethasone, hydroxycortisone, methylprednisolone, prednisone, and the antiviral drug remdesivir 22.

## 2. SARS-CoV-2 and Genomic Characterization

**Figure 2:**
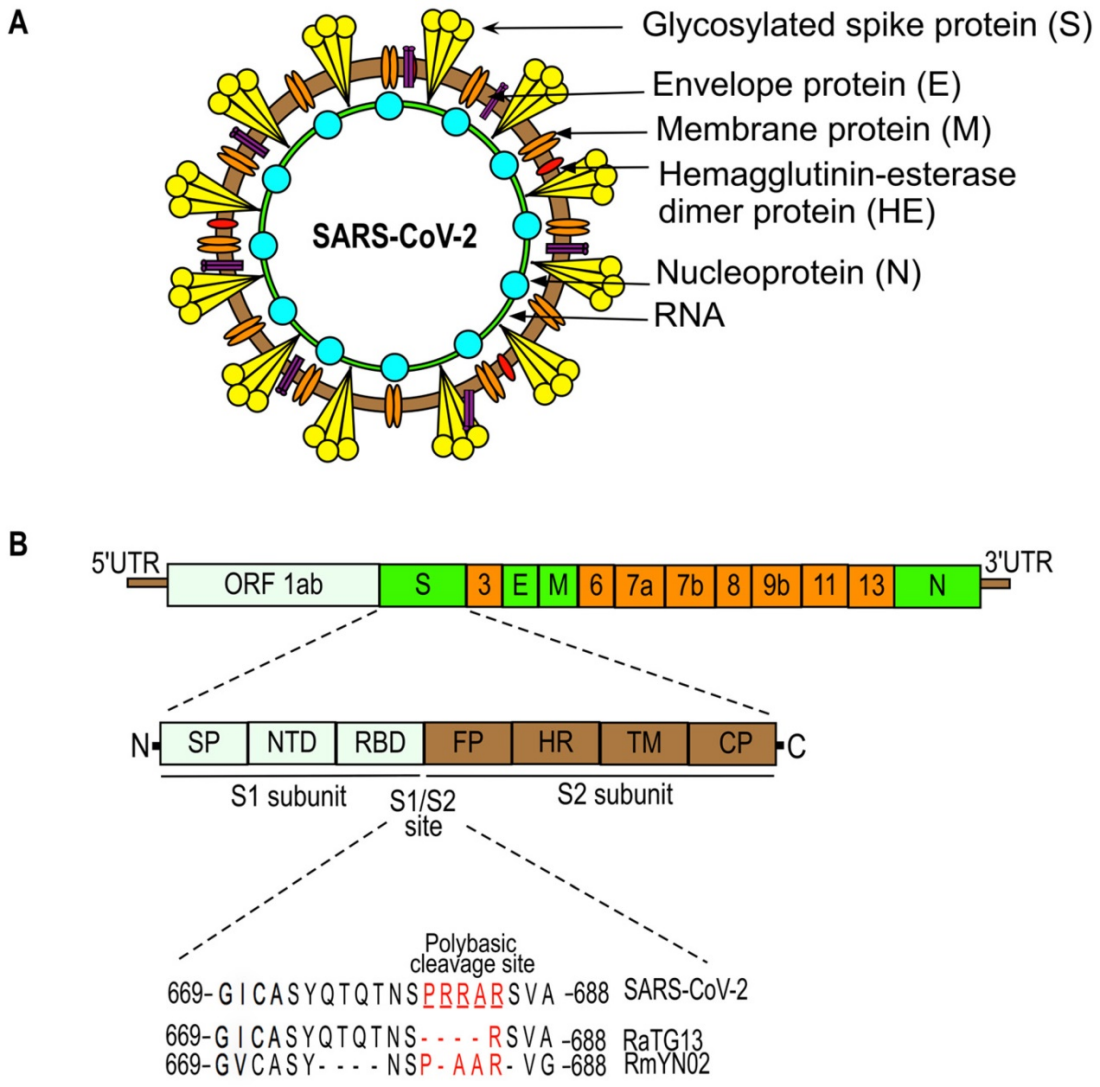
SARS-CoV-2 structure (a) and genome organization (b). The SARS-CoV-2 genome is comprised of: the 5'-untranslated region (5'-UTR); open reading frame (orf) 1a/b that encodes non-structural proteins (nsp) replicases; structural proteins including spike (S), envelop (E), membrane (M), and nucleoproteins (N); accessory proteins such as orf 3, 6, 7a, 7b, 8, 9b, 11, and 13; followed by 3'-untranslated regions (3'-UTR). Spike (S) protein has two functional domains, S1 (for attachment) and S2 (for fusion) and a polybasic cleavage site at the S1/S2 junction. Molecular characterizations of the S1/S2 cleavage site of SARS-CoV-2 and its closest relatives, RaTG13 and RmYN02 23, 25, 28, 30, 67, 137, 187, 188.

The genome organization of SARS-CoV-2 is given in Figure 2. SARS-CoV-2 (also named human 2019-nCoV HKU-SZ-005b, GenBank accession number MN975262) has a single-stranded RNA genome that is 29891 nucleotides in size and encodes 9860 amino acids 23. It consists of: a 5'-untranslated region (UTR); an open reading frame (ORF) 1a/b that encodes for nonstructural proteins (nsp) like replicases; structural proteins arranged in the order of Spike (S), Envelope (E), Membrane (M), and Nucleoprotein(N); and accessory proteins including ORF 3, 6, 7a, 7b, 8 and 9b followed by a 3'-UTR 23, 24. The spike protein of SARS-CoV-2, which facilitates the binding of the virus to its host cellular receptors, consists of two subunits, S1 and S2. The transmembrane S2 subunit is highly conserved and facilitates viral entry into target cells 25, 26. The S1 subunit consists of a signal peptide (SP), an N-terminal domain (NTD), and a receptor-binding domain (RBD). The RBD of SARS-CoV-2 is poorly conserved like other pathogenic human coronaviruses and is responsible for binding to the host angiotensin converting enzyme 2 (ACE2) receptor that facilitates the entry of the virus into target cells 25, 27. Most of the amino acid differences of the RBD are present in the external subdomain, whereas the core domain of the RBD is highly conserved. Six amino acids (L455, F486, Q493, S494, N501, and Y505) in the RBD domain are major determinants of efficient receptor binding of SARS-CoV-2 to the ACE2 receptor present on human cells. In addition, SARS-CoV-2 has four distinct amino acids located within the spike protein at the interface between the S1 and S2 fusion subunits. These unique sequences (underlined SPRRAR↓S) contain a cleavage site for the protease furin that plays an important role in the activation of the SARS-CoV-2 spike protein 25, 28, 29. The presence of multiple arginine residues at the S1/S2 site, also known as the poly-basic (furin) cleavage site, is found in all sequenced SARS-CoV-2 so far 30. The furin cleavage site is required for efficient proteolytic cleavage of the spike protein that is essential for S-protein-mediated cell-cell fusion and entry into human lung cells 28. Many CoVs require the host cell's endocytic machinery to enter and deliver their genome into host cells 31, 32. For example, mouse hepatitis coronavirus (MHV) enters the cells through clathrin-mediated endocytosis and later moves to lysosomes due to the fusion of endosomes with lysosomes. Lysosomal enzymes proteolytically process the viral S protein immediately upstream of the fusion peptide present in the S2 subunit (for reference, see Figure 2). This step is required for the fusion of viral membranes with endocytic vesicles resulting in the virus being released into the cell. In contrast, viruses like MERS-CoV do not require trafficking to lysosomes and processing by lysosomal proteases due to the presence of the furin cleavage site directly upstream of the fusion peptide at S protein. Moreover, the introduction of the furin cleavage site sequence just upstream of fusion peptide in MHV S protein abrogates the requirement of viral processing by lysosomal proteases for efficient infection. However, the virus still needs early endosomes for efficient infection, suggesting that the S protein is cleaved and activated in the early endocytic compartments by furin 31. In summary, CoVs that contain furin cleavage sequences immediately upstream of fusion peptide at S protein fuse in early endosomes in a furin-dependent manner, whereas those lacking these sequences are more likely to fuse in lysosomes after processing by lysosomes proteases 31.

Many proteases, including furin, type II transmembrane serine protease (TMPRSS2), PC1, trypsin, matriptase, cathepsin B, and cathepsin L, are reported to cleave the S1/S2 site and can potentially activate the SARS-CoV-2 S protein 28, 33. Furthermore, the S1/S2 site in SARS-CoV-2 contains a leading proline (underlined, SPRRAR↓S) that could affect the proteolytic cleavage and conformational changes in the spike protein, which in turn alters spike-host receptor engagement and the pathogenicity of the virus 33. It has been predicted that the leading proline residue allows the addition of O-linked glycans to neighboring residues that could create a mucin-like domain. The presence of these domains could shield the viral epitopes from host immune surveillance 34. Several viruses, including coronaviruses, utilize these mucin-like domains to evade the host's immune system, suggesting that SARS-CoV-2 might utilize this mechanism to positively influence virulence 35. However, Tse et al. have shown that a mutation eliminating a glycosylation site present very near to the furin-cleavage site in avian influenza virus H9N2 increases the transduction efficiency of the virus 36. Therefore, to experimentally determine the role of the predicted glycosylation near the S1/S2 site is important to decipher SARS-CoV-2 host cell infection and pathogenesis.

Due to extensive transmission in several countries, SARS-CoV-2 has accumulated genetic diversity over time 37. However, the acquired genetic diversity is moderate with an average pairwise difference of 9.6 SNPs between two viral genomes, suggesting a recent common ancestor. The analysis of genomic mutations present in the global population of SARS-CoV-2 reveals 198 recurrent mutations, out of which 80% produce no changes at the protein levels 37. The most recurrent mutations are found in three sites in the Orf1ab region (nucleotide positions 11,083, 13,402, 16,887) encoding Nsp6, Nsp11, Nsp13 proteins, and one in the spike protein region (21575) 37. The role of these mutations is not defined yet but it is reported that the Orf1ab region plays an important role in the virus' pathogenesis and adaptation in its host environment 38, 39. The sequence variability in the nsp2 gene of SARS-CoV-2 present in its Orf1ab region compared to SARS and bat SARS-like CoV could be responsible for some positive selection pressure that makes SARS-CoV-2 more contagious than others 40. The amino acid at position 501, which is position 321 of the Nsp2 protein and present in its endosome-associated-protein-like domain, has a glutamine residue, whereas the corresponding site in the SARS and bat SARS-like CoV has threonine and alanine residues, respectively. It could be possible that the presence of glutamine residue provides higher stability to the SARS-CoV-2 nsp2 protein due to its side chain length, polarity, and potential to form hydrogen bonds. Furthermore, this glutamine mutation in the Nsp2 protein of SARS-CoV-2 is similar to the one found in the Nsp2a region (Protein Data Bank: 3LD1) of avian infectious bronchitis virus which plays an essential role in virus pathogenicity 40. Pachetti et al. identified different point mutations in SARS-CoV-2 within different geographical areas, including Asia, Europe, and North America. The group reported three recurrent mutations in positions 3036 (present in nsp3 gene), 14408 (nsp12, also named as RdRp), and 23403 (spike protein) in Europe, and in positions 17746 (nsp13), 17857 (nsp13), and 18060 (nsp14) in North America. These mutations have not been detected in Asia which suggests that the virus is gaining different mutations over time within different geographical areas 41. Furthermore, a mutation in the RdRp (RNA-dependent RNA enzyme) of SARS-CoV-2 at position 14408 has been detected in the European population and is associated with a higher number of point mutations in those individuals 41. This makes sense because RdRp plays a key role in the replication and transcription cycle of the virus and mutations in its gene can contribute to impairing its proof-reading activity 42. In summary, the functional role of these mutations needs to be investigated further to understand if they are involved in virus pathogenicity and drug resistance. This is very important, especially as the SARS-CoV-2 pandemic is still accelerating fast in all regions of the world.

## 3. COVID-19 and Cancer

Cancer is a disease of abnormal cell growth anywhere in the body, with the potential to spread to other parts. The most commonly used cancer treatments work by killing or stopping the fast-dividing cancer cells from growing and spreading to other parts of the body. However, certain cancer treatments suppress the other rapidly-growing cells, like white blood cells (WBCs), including T and B lymphocytes in the bone marrow, and can weaken the immune system 43. Cancer itself can affect the immune system by spreading into the bone marrow 44. Therefore, people with a weak immune system have a higher risk of experiencing frequent infections and are more likely to get COVID-19. Studies show that COVID-19 increases complications and the overall risk of death in patients with cancer 19, 45-47. Compared to the general population, patients with cancer have a 3-fold vulnerability to death due to COVID-19 because their immune system can be weakened by cancer and its treatments 19. This study was performed on 105 cancer patients and 536 age-matched non-cancer patients with COVID-19. Patients with cancer have a relatively high death rate, high ICU admission rate, high chance of utilization of invasive mechanical ventilation, and high-risk of having critical symptoms compared to non-cancer patients due to COVID-19. Patients with hematological cancer, including leukemia, lymphoma, and myeloma, have the highest death rate, followed by lung cancer patients and esophageal cancer. Moreover, patients with stage IV metastatic cancer and COVID-19 led to overall high risks of death, ICU admission, development of severe conditions, and use of mechanical ventilation. Mechanical ventilation has been found to worsen outcome for patients because it is high stress and inefficiently delivers oxygen to compromised lungs. Further, patients undergoing different types of cancer treatments show disparities in response to COVID-19. Patients who received immunotherapy or surgery tend to have higher rates of death and higher chances of developing critical symptoms compared to those who received chemotherapy or radiotherapy 19.

A nationwide analysis of cancer patients with SARS-CoV-2 infection in China (18 out of 1590 COVID-19 cases) reveals that patients who had undergone chemotherapy or surgery had a higher risk of clinically severe events than patients who were not receiving these treatments for cancer 48. However, the interpretation of the findings in this study depended upon the small size of the cancer population (n=18), which could be a limiting factor for a solid conclusion. Furthermore, a retrospective analysis of 355 patients who died after SARS-CoV-2 infection in Italy revealed that 36% had diabetes, 30% had ischemic heart disease, and 25% had active cancer, whereas only 0.8% had no disease 49. A similar analysis by Trapani et al. on 909 patients who died from COVID-19 in Italy revealed that 17% were patients with cancer, which includes both cured and active cancer treatment recipients 50. A retrospective analysis of 1878 COVID-19 patients who visited a hospital in Madrid revealed that 2.4% were cancer patients, out of which 37.7% had lung cancer. Half of the lung cancer patients with COVID-19 had died (52.3%) compared to 10.2% deaths per 1878 total patients 45. Notably, dead lung cancer patients had a median age of 72 compared to the survivors' median age of 64.5 years.

**Figure 3:**
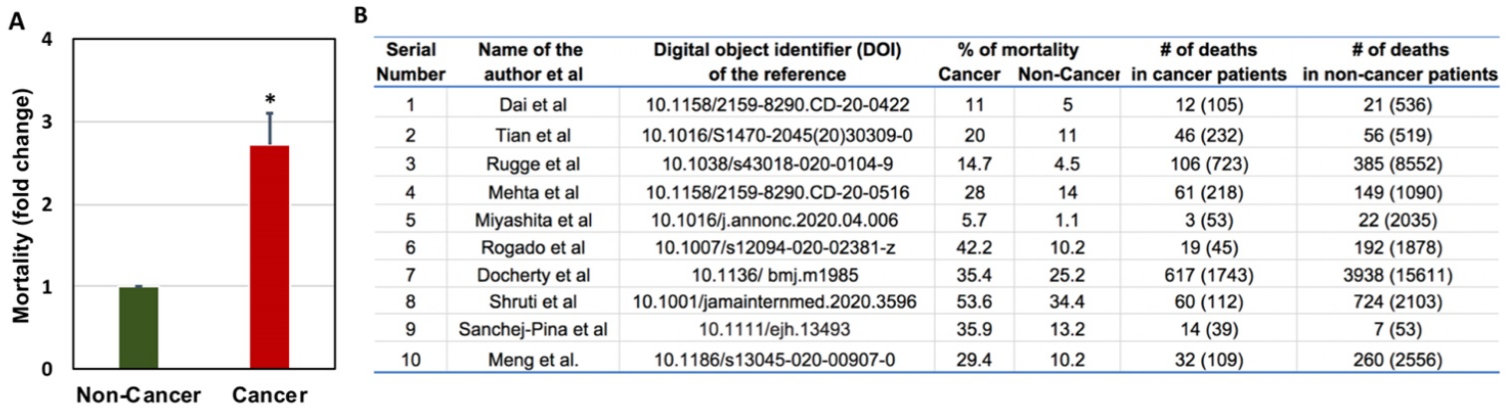
Comparison of COVID-19-related mortality in non-cancer and cancer patients (A) and the raw data used for the comparison of COVID-19-related mortality in non-cancer and cancer patients (B). Total number of patients are given in the brackets 19, 52, 58, 99, 181, 189-193.

**Figure 4:**
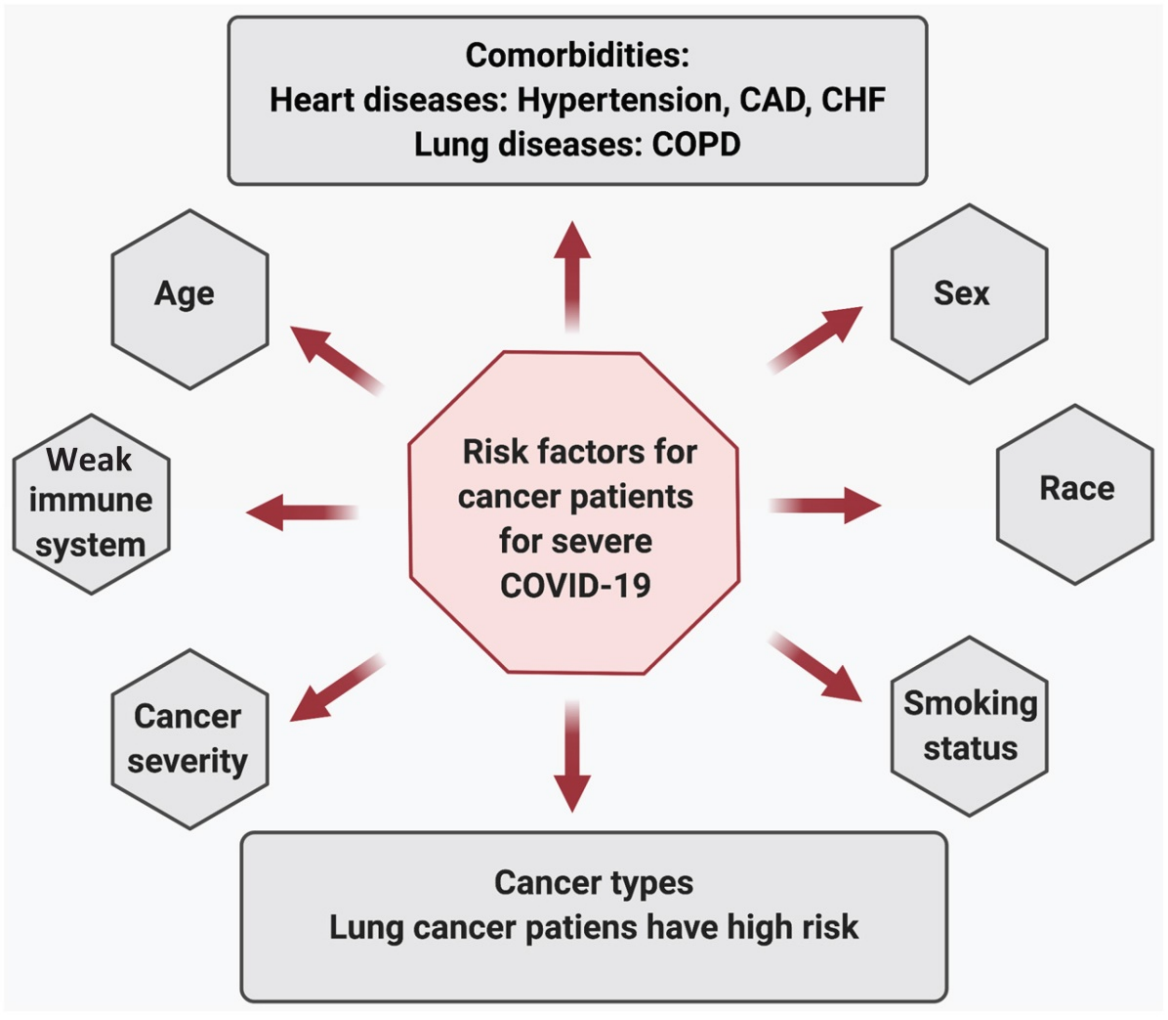
Risk factors associated with COVID-19 severity in cancer patients.

Zang et al. performed similar analyses where they studied 28 cancer patients who had a history of antitumor therapy out of 1276 total COVID-19 patients admitted to three hospitals in Wuhan, China. They found lung cancer as the most frequent cancer type, followed by esophageal cancer and breast cancer 46. Out of all the cancer patients, 53.6% had severe events (e.g., admitted to the ICU, required mechanical ventilation) and 28.6% died. This percentage is very high compared to the general population infected with SARS-CoV-2, where hospitalization rate is 0.16%, with the highest rates in people aged 65 years or older (0.3%) 51. In China, only 4.7% of cases required critical care and 2.3% of cases were fatal 46. The number of cancer patients death with COVID-19 when compare to non-cancer patients is depicted in Figure 3. The risk factors associated with COVID-19 severity in cancer patients is further presented in Figure 4.

**Risk factors associated with COVID-19 severity in cancer patients:** Severe COVID-19 illness and mortality in cancer patients are significantly associated with age, disease severity, multiple comorbidities, and habits like smoking status. A study conducted by Mehta et al. on 218 cancer patients with SARS-CoV-2 infection reveals that older age is significantly associated with an increase in mortality due to COVID-19 52. Patients with severe disease, including those who needed ventilator support or ICU care, had high mortality. The group did not find a statistical significance between advanced metastatic disease and death due to COVID-19. Also, patients undergoing chemotherapy or radiation therapy did not show a significant difference in COVID-related deaths than patients currently not under treatment. Co-morbidities, including heart diseases (hypertension, coronary artery disease [CAD], congestive heart failure [CHF]), and chronic lung disease increased the risk of COVID-19-related deaths in cancer patients. Additionally, when comparing alive and dead cancer patients due to COVID-19 revealed that post-SARS-CoV-2 infection, patients who eventually died had lower hemoglobin, higher WBC and neutrophil counts, and elevated inflammatory markers, including D-Dimer, lactate, and lactate dehydrogenase 52. Furthermore, compared with all COVID-19 cases or age and sex-matched non-cancer COVID-19 patients, cancer patients are dying at a significantly higher rate in all age groups, again suggesting that COVID-19 affects cancer patients much worse than the healthy population 52-54.

In lung cancer patients, COVID-19 severity was associated with age, smoking history, chronic obstructive pulmonary disease (COPD), hypertension, and CHF^55^ . Those who had reduced their smoking habits and had not being diagnosed with COPD or CHF had increased odds of recovery. In addition, elevated creatinine levels were associated with increased severity (ICU, intubation, or death) in cancer patients. However, patients who recently received chemotherapy or treatment with tyrosine kinase inhibitors did not show any difference in severity (hospitalization, ICU, intubation, or death) compared to other lung cancer patients 55.

In patients with thoracic malignancies (including non-small-cell lung cancer [NSCLC], small-cell lung cancer, mesothelioma, thymic epithelial tumors, and other pulmonary neuroendocrine neoplasms), age (>65 years), smoking status, receiving treatment with chemotherapy alone, and the presence of any comorbidities were associated with increased risk of death due to COVID-19 56.The risk factors associated with the worst overall survival in patients with haematological malignancies and COVID-19 (n=536) were older age, progressive disease status, diagnosis of acute myeloid leukemia, indolent non-Hodgkin lymphoma, aggressive non-Hodgkin lymphoma or plasma cell neoplasms, and severe or critical COVID-19. Furthermore, the patients were at a higher risk of mortality regardless of whether they had a recent disease or were on a specific therapy, or both. The mortality rate in patients with haematological malignancies and COVID-19 were high compared to the general population with COVID-19 and patients with haematological malignancies without COVID-19 57. Similar outcomes were reported by Sanchez-Pina et al., where COVID-19 patients with hematological malignancies (n=39) had a significantly higher mortality rate compared to non-cancer patients. The risk factors associated with mortality were age (>70 years) and the concentration of C reactive protein (>10 mg/dl). The active chemotherapy treatment and viral load at diagnosis were not predictors of the worst outcomes in these patients 54, 58.

Furthermore, in breast cancer patients, age (>70 years) and hypertension were significantly associated with COVID-19 severity, which included ICU admissions or death 59. In a cohort study of 1035 cancer patients (median age 66 years) with COVID-19 the most prevalent malignancies were found to be breast and prostate cancer 47. Thirteen percent patients had died within four weeks of COVID-19 diagnosis. Multiple prognostic variables were associated with COVID-19 related mortality in these cancer patients. Age (fatalities increased with age), male sex (more males had died or were admitted to the ICU than females), smoking status (more current or former smokers had died), number of comorbidities (a greater number of comorbidities associated with more death), types of malignancy (more solid tumor patients have died compared to those with hematological malignancy), and cancer status. However, race, ethnicity, obesity, types, or malignancy or cancer treatment were not associated with mortality 46, 47. Table 1 shows the effect of cancer treatment on COVD-19 severity in cancer patients. Figure 4 depicts the risk factors associated with COVID-19 severity in cancer patients.

In summary, cancer patients are more vulnerable to COVID-19 related illness and multiple risk factors are attributed to the disease's severity 60. Additionally, it seems that cancer treatment does not associate with COVID-19 severity, which could help cancer patient's well-being during this pandemic.

## 4. COVID-19, ACE2 receptor, TMPRSS2 and Cancer

The sequence similarities between RBDs of SARS-CoV-2 and SARS-CoV suggest that SARS-CoV-2 may utilize ACE2 as a cellular entry receptor present on human host cells 61. Hoffmann et al. reported that like SARS-CoV, SARS-CoV-2 entry into host cells depends upon the binding of viral spike (S) protein to ACE2 receptors present on the host cells 62. ACE2 is a zinc-dependent metalloprotease that primarily regulates the renin-angiotensin-aldosterone (RAAS) pathway 63. RAAS is a multi-hormonal system that regulates blood pressure and fluid balance in the body. ACE2 receptors are present all over the body in many cell types, but their quantity can vary among individuals and cell types 64-66. The presence of ACE2 receptors on lungs, which are the primary site of infection by SARS-CoV-2, aids virus entry into the lungs 62, 67. The S1 domain of the viral spike protein mediates receptor binding, whereas the S2 domain allows viral membrane fusion with the host cells. SARS-CoV-2 requires endocytic protease-primed cleavage event at its S1/S2 site for fusion 25, 62, 68. The presence of multiple arginine residues at the S1/S2 site undergo proteolytic processing on the surface of human cells, which exposes fusion sequences of spike protein and allows the virus to fuse with the host membrane. Host cells endosomal cysteine proteases, including TMPRSS2 and cathepsin B and L, catalyze this proteolytic activation 33, 62. Unlike SARS-CoV, SARS-CoV-2 spike protein is also cleaved and pre-activated during virus packaging at the furin cleavage site at S1/S2, reducing its dependence on host cell proteases for entry 68. The spike protein pre-activation at the furin cleavage site and proteolytic activation by host cell surface proteases have cumulative effects on virus infection which could make SARS-CoV-2 more infectious than its relative viruses (SARS-CoV and influenza).

It is believed that SARS-CoV-2 infection decreases ACE2 receptors activity because viruses occupy these receptors, making them less available for other functions. One of the important functions of ACE2 receptors is the degradation of Ang II protein, resulting in the formation of vasodilator angiotensin 1-7 (Ang 1-7) 69. Accumulation of Ang II induces inflammation by activating a number of inflammatory monocytes, C-reactive protein, and generating reactive oxygen species (ROS) 70. ACE2 utility during SARS-CoV-2 infection induces a robust inflammatory response that results in severe inflammation in the body and life-threatening symptoms. A study in previously healthy children and adolescents infected with SARS-CoV-2 shows that multisystem inflammatory syndrome due to infection is associated with serious and life-threatening illness 71 . Low ACE2 activity has been detected in many cancers when compared to healthy individuals 72-74. ACE2 overexpression inhibits tumor cell proliferation, invasion, epithelial to mesenchymal transition (EMT), and metastasis in various cancer types, suggesting its anti-tumor roles in various cancers types 75, 76. In addition, ACE2 expression positively correlates with low EMT scores in aerodigestive and respiratory cancer cell lines and in normal and cancer patients 77. The decrease in ACE2 expression in cancer patients with COVID-19 might play a critical role in promoting tumor phenotypes that further aggravate the disease. SARS-CoV-2 infection in lung cancer cell lines reduces ACE2 expression and upregulates Zinc Finger E-Box Binding Homeobox 1 (ZEB1). ZEB1 promotes EMT induction in these cells by negatively regulating ACE2, suggesting that SARS-CoV-2 infection might shift cells to a more mesenchymal phenotype 77. The infected cells show decreased dependence on glutamine synthesis, an important event in EMT 77. ACE2 expression positively correlates with tumor infiltration and favorable prognosis in UCEC (uterine corpus endometrial carcinoma) and KIRP (kidney renal papillary cell carcinoma) 78.

The ACE2 promoter is hypomethylated in these cancers and the level of hypomethylation is lower in high grade and serous tumors than control tumors 78. DNA hypomethylation is a common event in cancer and has been linked with the activation of genes during tumor progression 79. The reduction in ACE2 expression due to SARS-CoV-2 infection may hamper its tumor-suppressive and immune-activating effects in UCEC and KIRP which may worsen the prognosis of COVID-19 in these patients. Furthermore, the high expression of host cell protease TMPRSS2 promotes SARS-CoV-2 fusion in both localized and metastatic prostate cancers. TMPRSS2 is regulated by the androgen receptor in prostate development, but its aberrant activation leads to prostate cancer 80, 81.

Androgen deprivation therapy (ADT) is the primary treatment for prostate cancer. ADT decreases the levels of TMPRSS2 in prostate cancer patients 82. Montopoli et al. reported that prostate cancer patients receiving ADT are partially protected from SARS-CoV-2 infection 83. The group studied 5273 patients receiving ADT and found that they had a significantly lower risk of SARS-CoV-2 infection compared to patients who did not receive ADT (n=37161, OR 4.05; 95%Cl 1.55-10.59) or patients with other types of cancer (n=84934, OR 4.86; 95% Cl 1.88-12.56). This could be due to less TMPRSS2 expression in these patients that decreases vulnerability to SARS-CoV-2 83. This can support the notion of why males are more vulnerable to COVID-19 compared to females and children 84. Genetic variants in the androgen receptor may be linked to race gaps in COVID-19 related infections and deaths. ACE2 and TMPRSS2 are abundantly expressed in intestinal epithelial cells and can promote SARS-CoV-2 infection in human small intestinal enterocytes 85, 86. Further, high expression of ACE2 in the male urogenital system organs, including the prostate, and the overexpression of androgen receptor-regulated TMPRSS2 in prostate cancer patients, could increase their susceptibility to SARS-CoV-2 87-89. In patients with irritable bowel syndrome (IBS), the ACE2 and TMPRSS2 expressions in the colonic mucosa are not increased compared to control patients without IBD. Similarly, the expression of two genes is not influenced in the presence of inflammation in ileum or colon, suggesting that IBD patients are not at increased risk of gastrointestinal infection due to SARS-CoV-2 85.

## 5. Inflammation and immunity in COVID-19 cancer patients

SARS-CoV-2 has been transmitted almost everywhere in the world and the total number of COVID-19 cases continue to rise 90. The number of cases and fatalities among older people (>65 years) are higher, and 8 out of 10 people who died due to COVID-19 in the U.S. were aged 65 or older 91, 92. The weaker immune system and the presence of multiple comorbidities in elderly patients increase the risk of severe illness from COVID-19. Advanced age is an important risk factor associated with many cancer types and 25% of new cancer cases diagnosed are in people age 65 to 74. Another 24% and 19.6% are in the age groups 55-64 and 75-84, respectively 93. This means that cancer is an age-related disease in most cases, and the risk of getting cancer increases with age. COVID-19 negatively affects vulnerable cancer patients to a greater extent compared to the rest of the population because of their age and suppressed immune system due to cytotoxic therapies or by the cancer itself.

The main route of SARS-CoV-2 infection is through respiratory droplets 94. The ACE2 receptors present on human respiratory epithelia facilitates SARS-CoV-2 entry into host cells 62, 95. SARS-CoV-2 entry into respiratory epithelia leads to virus replication and multiplicity of infection. Although the overall expression of ACE2 receptors is low in respiratory epithelia, it is expressed by multiple epithelial cell types across the airway and alveolar epithelial type II cells, a key defender against foreign pathogens in the lungs 96. Notably, nasal epithelial cells show the highest expression of ACE2 receptors among all cells in the respiratory system 96. Infection with SARS-CoV-2 leads to an aggressive inflammatory response that is actually the reaction of the immune system 97. Inflammation of air sacs in the lung tissues leads to pneumonia, which causes shortness of breath, cough, fatigue, and fever. Pulmonary pathology of two lung cancer patients (age 84 and 73) who recently underwent lung lobectomies and had SARS-CoV-2 infection showed edema, vascular congestion, and focal fibrin clusters mixed with mononuclear inflammatory cells and multinucleated giant cells in the air sacs 98. Compared to patients without cancer (n=519), patients with cancer (n=232) were more likely to have dyspnea (difficulty in breathing, n=63[27%] vs n=89 [17%], cancer vs non-cancer, p=0.0022) and expectoration (ejecting phlegm or mucus, n=52[22%] vs n=83[16%], cancer vs non-cancer, p=0.044), but less likely to have sore throat and coryza (inflammation of mucus membrane in the nose) due to COVID-19 99. Other common symptoms, including fever, dry cough, and fatigue, were not significantly different in cancer vs non-cancer patients. In addition, computed tomography (CT) scans of cancer patients show ground-glass opacity (148 out of 195 [76%] vs 183 out of 301 [61%], cancer vs non-cancer, p=0.0007) and patchy shadows (126 out of 195 [65%] vs 152 out of 301 [50%], cancer vs non-cancer, p=0.0027) more frequently compared to non-cancer patients. The levels of pro-inflammatory cytokines, including TNF-α (n=89 vs n=336, 8.7 vs 6.9 pg/ml, p=0.004, cancer vs non-cancer patients), IL-6 (n=138 vs n=350, 12.8 vs 4.9 pg/ml, p<0.0001, cancer vs non-cancer), and IL-2R (n=79 vs n=340, 615 vs 535 U/ml, p=0.012, cancer vs non-cancer), and infection-related biomarkers, like procalcitonin (n=161 vs n=251, 0.3 vs 0.1 ng/ml, p=0.0041) and C-reactive protein (n=91 vs n=246, 46.4 vs 40.7 mg/L, p=0.047), were higher in cancer patients. Moreover, cancer patients had a significant reduction in lymphocytes, including CD4+T cells (n=37 vs n=82, 370 vs 625.5 counts/μl, p<0.0001, cancer vs non-cancer) and CD8+T cells (n=43 vs n=82, 206 vs 305.0 counts/μl, p<0.0081, cancer vs non-cancer) 99. This makes sense because tumor cells induce immune system dysfunctions characterized by impaired T cell-mediated cytotoxicity and reduced T cell proliferation. This could be a risk factor associated with cancer patients that make them more vulnerable to SARS-CoV-2 infection.

A study by Diao et al. showed that T cell counts were significantly reduced and functionally exhausted in COVID-19 patients 100. The CD8+ and CD4+ T cell numbers were negatively correlated with COVID-19 patient survival. In addition, the higher percentage of CD8+ and CD4+ T cells showed upregulated programmed cell death protein 1 (PD-1) and T-cell immunoglobulin and mucin-domain containing-3 (Tim-3). Some cancer cells have a large amount of PD-L1, a transmembrane protein that acts as a ligand for PD-1. The binding of PD-L1 to PD-1 activates the down-stream signaling of PD-1 receptor in T cells which inhibits their proliferation and cytotoxic activity 101. Hence, high expression of PD-L1 in cancer patients could make them more susceptible to foreign pathogens like SARS-COV-2.

Furthermore, cancer patients are more likely to have multiple organ damage compared to non-cancer patients after SARS-CoV-2 infection 99. Cancer patients showed significantly higher levels of alanine transaminase, lactate dehydrogenase, and albumin/globulin ratio 99. In haemato-oncology patients, elevated levels of C-reactive protein and hypoxia were predictive of poor outcomes in COVID-19 patients, whereas hemoglobin concentration, platelets count, and neutrophil/lymphocyte ratio did not show a significant association with disease severity 60. Analysis of COVID-19-related anxiety in breast cancer patients showed that SARS-CoV-2 infection could affect patients' decision-making process 102. Vanni et al. analyzed the decision-conflict for non-metastatic breast cancer patients making choices about procedure and surgery 102. The group divides patients who have suspected breast lesion (n=82) or breast cancer (n=78) into two groups: one who visited the hospital before COVID-19 era (pre-COVID, n=43 and 41 for suspected breast lesion and breast cancer, respectively) and the other after COVID-19 (post-COVID, n=39 and 37 for suspected breast lesion and breast cancer, respectively). Procedure refusal and surgical refusal rates among post-COVID cancer patients were significantly higher than pre-COVID ones, suggesting that fear and anxiety of SARS-CoV-2 infection could impact cancer patients' decisions on treatment refusal 102.

## 6. Treatment impact in COVID-19 patients with cancer

There is no specific antiviral treatment for COVID-19 and most people have recovered at home. The treatment or therapies that are given or under investigation includes non-invasive or invasive mechanical ventilation to prevent respiratory impairment 103, drugs including corticosteroids (e.g., dexamethasone) 104, anti-malarial (e.g., hydroxychloroquine [HCQ]) 105 and anti-viral (e.g., remdesivir, favipiravir, lopinavir, or ritonavir) 14, 106, convalescent plasma therapy 107, and anti-inflammatory antibodies (e.g., anti-IL-6 receptor antibody tocilizumab) 108. The treatment strategies used against COVID-19 patients with cancer are shown in Table 2.

**HCQ controversy:** HCQ is an FDA-approved drug to treat or prevent malaria as well as autoimmune conditions such as chronic discoid lupus erythematosus, systemic lupus erythematosus in adults, and rheumatoid arthritis 109. Although the precise mechanism of action is unknown, HCQ acts as a lysosomotropic agent, raises intralysosomal pH, and impairs the autophagy/lysosomal degradation pathway 110. HCQ may suppress immune function by interfering with the processing and presentation of antigens and the production of cytokines 111, 112. Also, HCQ can inhibit the replication of SARS-CoV-2 *in vitro* 113. Some observational studies have suggested no benefits of HCQ for the treatment of COVID-19, whereas some have shown an improved outcome. A study by Luo et al. showed that HCQ administration to COVID-19 patients with lung cancer (n=35) did not improve outcomes, including ICU admissions, intubation, or death. Although the severity of COVID-19 is higher in lung cancer patients, the majority of patients (65%) have recovered 55. Another study looking for the effect of HCQ and azithromycin on severe COVID-19 patients with cancer revealed that combination treatment of these drugs increases the mortality in patients 47. Eighty-nine cancer patients were given HCQ, out of which 11 (12%) died, 18 (20%) were admitted to the ICU, and 14 (16%) required mechanical ventilation. In azithromycin alone treatment (n=93), 12 (13%) died, 15 (16%) were admitted to the ICU, and 14 (15%) required mechanical ventilation. The patients treated with a combination of HCQ and azithromycin (n=181), shows 45 (25%) death, 53 (29%) were admitted to the ICU, and 51 (28%) required mechanical ventilation compared to untreated patients (n=486), where only 41 (8%) have died, 39 (8%) were admitted to the ICU, and 29 (6%) required mechanical ventilation. This showed that taken alone or in combination, these drugs did not improve clinical outcomes and may be toxic to already vulnerable cancer patients 47. However, it is noted that in this study the drugs were given to severe COVID-19 patients compared to controls, raising doubt that the treatment is toxic to the patients. But HCQ or azithromycin or their combination does not improve COVID-19 related illness in cancer patients. An observational study on 2186 cancer patients with COVID-19 found that patients who received HCQ in combination with: azithromycin (n=203, 23%), or azithromycin plus high dose corticosteroids (n=24, 3%) or tocilizumab (n=18, 2%), or tocilizumab plus azithromycin (n=18, 2%) had increased risk of 30-day all-cause mortality compared to matched or unmatched positive controls (drugs treatment without HCQ) or negative controls (untreated). However, HCQ only treatment (n=179, 21%) was not associated with increased risk compared to positive or negative controls. The majority of patients in this study had a solid tumor (n=1781, 81%), of which breast cancer was the most common (n=455, 21%) followed by prostate (n=368, 17%) and gastrointestinal tumors (n=290, 13%) 114. Similar findings were seen in another observational study including 1376 COVID-19 patients examining the effect of HCQ on intubation or death 115. Out of 1376 patients, 811 (109 were cancer patients, 13.4%) were given HCQ (600 mg twice on day 1, then 400 mg daily for 5 days) with a median follow up of 22.5 days. HCQ treated patients were severely ill and did not show significant differences between HCQ use and intubation or death (hazard ratio [HR], 1.04; 95% confidence interval [CI], 0.82 to 1.32) compared to those who did not receive HCQ 115. Furthermore, a retrospective multicenter cohort study involving 1438 COVID-19 patients in 25 hospitals of the New York metropolitan region was performed to determine the clinical benefits of HCQ, with or without azithromycin, and found no significant difference in mortality for patients receiving these drugs compared to patients receiving neither drug 116. Out of 1438 COVID-19 patients, 735 received HCQ and azithromycin, 271 received HCQ, 211 received azithromycin and 221 received neither drug. After adjustment for demographics, specific hospital, preexisting conditions, and illness severity on these patients, there were no significant differences in mortality between patients receiving HCQ + azithromycin (adjusted HR, 1.35 [95% CI, 0.76-2.40]), HCQ alone (adjusted HR, 1.08 [95% CI, 0.63-1.85]), or azithromycin alone (adjusted HR, 0.56 [95% CI, 0.26-1.21]), compared with neither drug. Furthermore, patients receiving HCQ + azithromycin were more likely to have cardiac arrest than other treatment groups 116. These findings are supported by a multi-center, randomized, controlled trial including 4716 COVID-19 patients, out of which 1561 were given HCQ and 3155 received usual care alone.

In the HCQ group, 26.8% patients met the primary outcome of 28-day mortality, whereas in the usual care group, 25% patients met that criteria. The difference between the two groups was statistically insignificant (p=0.18) with a rate ratio (HCQ treated/usual care) of 1.09 (95% CI 0.96 to 1.23). Additionally, the HCQ treated group took a longer time to be discharged alive from a hospital compared to usual care within 28 days (HCQ treated vs. usual care, discharged n=941, 60.3% vs n=1982, 62.8%) [95% CI 0.85 to 0.99] (ClinicalTrials.gov Identifier or Trial identifier: TI: NCT04381936) 117. Moreover, Prevention and Early Treatment of Acute Lung Injury (PETAL) Clinical Trials Network of National Heart, Lung, and Blood Institute (NHLBI) of the NIH conducted a clinical trial to evaluate the safety and effectiveness of HCQ for hospitalized COVID-19 patients (TI: NCT04332991). The study was named Outcomes Related to COVID-19 treated with HCQ among In-patients with symptomatic Disease, or ORCHID Study, and aimed to enroll more than 500 hospitalized adults with COVID-19. The study concluded that HCQ did not provide an additional clinical benefit compared to placebo control for COVID-19 treatment. The NIH terminated this study, concluding that HCQ treatment neither harms nor provides benefit to hospitalized COVID-19 patients. There were more than 470 patients enrolled at the time of study closure 118.

In contrast, a study showed that the combined treatment of HCQ with antibiotic azithromycin significantly improves the outcome of COVID-19 lung cancer patients (1 dead out of 8 patients, OR 0.04, CI 0.01-0.57, p=0.018) suggesting that it could be a better therapeutic option 45. The age of the dead person was 72 years vs the survivors' average age of 64.5 years (p=0.12). Other reports have supported this with similar findings of reduction in mortality in COVID-19 patients treated with HCQ alone (162/1202, 13.5% [95% CI: 11.6%-15.5%]) or in combination with azithromycin (157/783, 20.1% [95% CI: 17.3%-23.0%]) when compared to those not receiving HCQ (108/409, 26.4% [95% CI: 22.2%-31.0%]) or receiving azithromycin only (33/147, 22.4% [95% CI: 16.0%-30.1%]). The mortality HR in the HCQ group and HCQ + azithromycin group is decreased by 66% and 71%, respectively 105. There is an ongoing trial on cancer patients infected with COVID-19 in France investigating HCQ and azithromycin in the treatment of COVID-19. HCQ (800 mg) is given on day one followed by 400 mg/day for four days, whereas 500 mg of azithromycin is given on day one and then 250 mg/day for four days (TI: NCT04341207). In summary, a majority of studies have shown that HCQ did not prevent COVID-19 or reduce the risk of death among hospitalized patients with COVID-19. Therefore, the NIH recommends against the use of chloroquine (CQ) or HCQ alone or in-combination with azithromycin for the treatment of COVID-19 in hospitalized and non-hospitalized patients except in clinical trials 119.

**Interleukin (IL) inhibitors:** One of the hallmarks of SARS-CoV-2 infection is pathological inflammation that is associated with disease severity and death in patients 120. COVID-19 patients have increased amounts of proinflammatory cytokines, including IL-6, IL1β, IFNγ, IP10, and MCP1 in serum. The higher concentrations of GCSF, IP10, MCP1, MIP1A, and TNFα are found in patients that require ICU admission after COVID-19, suggesting that the cytokine storm, where the body starts to attack its cells, is associated with disease severity 15. Trials to block this cytokine storm have been going on across the world and have shown some clinical benefits 121, 122. IL-6 inhibitors, including antibodies tocilizumab and sarilumab, that block the IL-6 receptor are in use as investigational therapies for COVID-19 treatment. Tocilizumab shows some significant improvement in COVID-19 patients experiencing cytokine storm 121, whereas sarilumab trials are underway (TIs: NCT04315298 and NCT04327388). A retrospective cohort study by Guaraldi et al. found that intravenous or subcutaneous treatment of tocilizumab could reduce the risk of invasive mechanical ventilation or death in COVID-19 patients. However, this effect was not seen in cancer patients infected with COVID-19. The study compared the effect of tocilizumab (n=2) and no tocilizumab treatment (n=8) on cancer patients. The authors did not find any significant clinical outcomes in the two groups (p=0.38). Both groups underwent standard treatment, including oxygen supplementation, hydroxychloroquine, azithromycin, antiretrovirals, and low molecular weight heparin treatment. The authors did not specify which type of standard treatment was given to each cancer patient 123.

A similar study comparing the effect of tocilizumab on treating COVID-19 symptoms in two cancer patients showed no improvement compared to no tocilizumab treatment 124. In contrast, Michot et al. reported successful treatment of a COVID-19 patient, a 42-year male recently diagnosed with metastatic sarcomatoid clear cell renal cell carcinoma. He was first given lopinavir-ritonavir (400-100 mg) orally on day 7 (started from the day when he came to the hospital with COVID related symptoms) for five days. His condition did not improve, and on day 8 he was treated with two 8 mg/kg doses of tocilizumab, 8 hours apart. He started experiencing clinical improvement, became afebrile, and on day 19 he had partial regression of the pulmonary infiltrates and a decrease in C-reactive protein concentration from 225 mg/l to 33 mg/l in 4 days. The amount of circulating lymphocytic subpopulations and CD4+CD25+ cells remains unchanged before and after tocilizumab treatment. The patient was clinically fully recovered from COVID-19 symptoms 108. Again, a very small sample size can compromise the conclusions drawn from the studies and we need a cohort study on cancer patients evaluating the effectiveness of IL-6 therapy against COVID-19 symptoms. IL-6 inhibition therapies have been explored as therapeutic targets in patients with cancer. However, the lack of effect of these therapies due to tumor cell plasticity, and other growth factors activating pathways like those triggered by IL6, raises doubt about the efficacy of IL-6 inhibitors. Furthermore, IL inhibitors protect patients with chronic inflammatory disorders, like asthma, from COVID-19 as anti-inflammatory drugs help cancer pain management 125, 126. COVID-19 patients who had severe eosinophilic asthma and underwent treatment with monoclonal antibodies against IL-5, including reslizumab and benralizumab, showed less severe symptoms of COVID-19 127, 128. Renner et al. did a case study on a 41-year old male patient diagnosed with COVID-19 who had severe eosinophilic asthma for nine years. The patient underwent treatment with monoclonal antibodies against IL-5, including reslizumab and benralizumab from the past four years. The patient did not develop severe exacerbation due to COVID-19 and become free from symptoms after a week. Notably, this patient always needed oral corticosteroids for viral infections until anti-IL5/IL-5R antibodies treatment was started 127. A similar finding was reported by Ismael García-Moguel et al. in the context of COVID-19 patients (n=2) with severe eosinophilic asthma who underwent benralizumab treatment. After being diagnosed with COVID-19, one patient was treated with systemic corticosteroids and the other with azithromycin, HCQ, and amoxicillin-clavulanic acid. Both patients responded well to the therapy and recovered from COVID-19. Notably, SARS-CoV-2 is a respiratory virus, and it is reasonable to expect greater disease severity in patients with moderate to severe asthma. In contrast, the patients who underwent treatment with anti-IL5/IL-5R antibodies for asthma responded well to COVID-19 treatment. This suggests that anti-IL5/IL-5R treatment may have some protective effect against COVID-19, however, we cannot rule out the possibility that COVID-19 medications were also given to these patients. Regardless, these findings encourage the continuation of anti-IL5/IL-5R treatment in patients with asthma during the COVID-19 era 128.

***Antiviral protease inhibitors:*** Another treatment used for COVID-19 is a combination of the protease inhibitors lopinavir and ritonavir approved for the human immunodeficiency virus (HIV) infection 129, 130. However, this combination does not significantly benefit COVID-19 patients (n=199). Five cancer patients were given lopinavir and ritonavir treatment at 400 mg and 100 mg doses, respectively, twice a day for 14 days in addition to standard care and were compared with standard care of only patient (n=1). The treatment did not change the clinical improvement in patients, reduce mortality, or decrease viral RNA load 130. However, the combination of lopinavir-ritonavir with interferon-beta-1b and the anti-viral drug ribavirin significantly diminished SARS-CoV-2 load and the reduced median time from start of the study treatment to negative nasopharyngeal swab from 12 days to 7 days compared to lopinavir-ritonavir treatment 131.

A study by Zhang et al shows that cancer patients who had anti-tumor therapy are less likely to respond to COVID-19 treatment and have more risk of developing severe events 46. In this retrospective cohort study of 28 SARS-CoV-2-infected cancer patients, 20 patients (71.4%) were prescribed at least one antiviral agent, including arbidol (n=14, 200 mg orally, three times a day), lopinavir and ritonavir combination (n=10, 400 mg and 100 mg orally, twice a day), ganciclovir (n=9, 500 mg, intravenous [i.v] drip, twice a day), and ribavirin (n=1, 500 mg, i.v drip, twice a day), 9 patients (32.1%) were administered combinations of antiviral agents, 15 patients were given systemic corticosteroids (n=15, 53.6%), and 10 patients were given immunoglobulin treatment. Systemic corticosteroid treatment was more frequent in patients with severe events, which included admission to the ICU or the use of mechanical ventilation. Eight patients (28.6%) died during the treatment and ten patients (35.7%) with free of COVID-19 symptoms were discharged from the hospital with a median stay of 19 days (interquartile range=16.0-28.5). The severity of the disease was associated with tumor stage and anti-tumor treatment. Upon comparison with patients who received anti-tumor treatment within 14 days, including chemotherapy (n=3), radiotherapy (n=1), targeted therapy (n=2), and immunotherapy (n=1), 5 out of 6 patients (83.3%) developed severe events compared to 10 out of 22 (44.4%) patients who did not receive this treatment (HR=4.079, 95% CL 1.086-15.322, p=0.037). Additionally, when comparing stage IV to non-stage IV cancer patients undergoing COVID-19 treatment, 7 out of 10 (70%) patients developed severe complications vs 8 out of 18 (44.4%) non-stage cancer patients. Moreover, upon comparing patchy consolidation vs no patchy consolidation on CT scan, 11 out of 13 (84.6%) developed severe events versus 4 out of 15 (26.7%) (HR 5.000, 95% CI 1.576-15.861, p=0.006) 46. To summarize these studies, we conclude that some SARS-CoV-2 infected cancer patients respond well to certain treatments whereas others do not respond. However, some critical factors may play a role in this discrepancy, including tumor type, tumor stage, tumor treatment, delayed admission time, age, comorbidities, and symptoms like lung consolidation.

***Corticosteroids:*** It is plausible that special precautions need to be taken when treating COVID-19 in people with cancer because they are at higher risk for more severe symptoms that could lead to death. Similarly, certain COVID-19 treatments could be effective against the normal population but need careful consideration when applied to cancer patients. For example, dexamethasone treatment is recommended by the NIH and showed promising results in lowering 28-day mortality in severe patients including those who were receiving either invasive mechanical ventilation or oxygen support 132. Dexamethasone is recommended in cancer patients to reduce inflammation and lower the body's immune response 133. A study by Cook et al. showed that dexamethasone co-medication in cancer patients depletes the CD4+ and CD+ T cell population and activates immunosuppressive regulatory T cells. Reduction in CD4+ and CD+ T cell population also been seen in COVID-19 patients 134. In addition, T cells from COVID-19 patients express significantly higher levels of PD-1, which is associated with T cell exhaustion and apoptosis 100. Therefore, treating cancer patients, especially those who undergo cancer treatment, for COVID-19 with drugs having immunoinhibitory effects could increase the risk of severe complications and opportunistic infections. Furthermore, cancer patients who had treatment with immune checkpoint inhibitors could be associated with more severe disease related to COVID-19 treatment 135. Immune checkpoint inhibitors prevent signals from being sent to T cells and hyperactivate them to fight cancer. Unfortunately, immune hyperactivation is associated with cytokine storm, a symptom that is often seen in COVID-19 patients, leading to acute respiratory distress syndrome and multiple organ failure 136. Therefore, a synergy between immune checkpoint inhibitors and COVID-19 in cancer patients cannot be ruled out and the choice of drugs should be considered with utmost care. This hypothesis is supported by Robilotti et al. who found that immune checkpoint inhibitors treatment was associated with hospitalization and severe respiratory illness due to COVID-19 in cancer patients 135.

***ADT:*** ADT or related therapies for prostate cancer treatment seem to play a protective role against SARS-CoV-2. COVID-19 patients who had prostate cancer and received ADT for cancer treatment had a significant, four-fold reduced risk of getting COVID-19 compared to patients who did not receive ADT 83. Based on these findings, anti-ADT therapies are in clinical trials against COVID-19 (TI: NCT04446429). In addition, drugs that target TMPRSS2 expressions, such as Nafamostat (TI: NCT04473053) and bromhexine (TI: NCT04355026), are also under investigation for COVID-19 treatment.

## 7. Cancer patients care during COVID-19 pandemic

From the above discussion, it is clear that people with cancer who are being treated are at higher risk of developing severe COVID-19 symptoms that could lead to death. Cancer patients often require clinic visits for follow-up medical care, which could increase the risk of hospital-acquired infections during the COVID-19 era, making these immunocompromised patients more vulnerable to the infection.

**Basic recommendations:** The Centers for Disease Control and Prevention (CDC) in the U.S. recommend some guidelines for cancer patients, which they consider a high-risk population, and their caregivers to protect them from COVID-19. The basic recommendations are the same as what they suggested to the general public, which includes watching out for fever (38ºC or higher), knowing the signs and symptoms of infection, cleaning your hands often, avoiding touching one's face, using a face covering, avoiding people as much as possible, and keeping a distance of at least 6 feet (2 m) 137. Cancer patients are advised to get extra necessary medications in case they need to stay home for a long time and to call their doctor's office several days ahead of their appointment to make sure of the doctor's availability 138. Furthermore, cancer patients are advised to minimize hospital visits to prevent them from unnecessary infections and are requested to consider delaying treatments if their cancer responds well to the treatment 139. The patients need to contact their cancer care team for suggestions and recommendations related to managing this disease during the pandemic. Doctors are advised to discuss the benefits and risks of current cancer therapy with their patients during the COVID-19 pandemic. As discussed above, studies show that cancer patients who underwent cancer treatment and tested positive for COVID-19 have more severe outcomes compared to non-treated patients 19, 99, 135. Patients undergoing a different type of cancer treatment show different outcomes related to COVID-19 severity. It is important that patients or their caretakers should know the risk factors associated with COVID-19 severity. Doctors should stay up to date on COVID-19 research to make better decisions regarding treatment recommendations and patient health care. Effective communication among healthcare professionals, patients, and their caretakers is the key to redesign health care and weighing the benefits of care against the 'cost of social contact' during this pandemic.

**Treatment challenges:** Due to the highly contagious nature of COVID-19, its spread in the general public is very rapid. This has overwhelmed hospitals and put strain on healthcare workers 140. It is highly likely that newly diagnosed and existing patients with cancer might not get treatment on time. Additionally, mental stress about getting the infection during this infectious disease outbreak could drastically impact cancer patients' decisions to go to the hospital for follow up visits and treatment. The risk of not getting treatment on time, cancer-related psychological stress and distress, uncertainty, and social isolation without knowing when the pandemic will end could negatively affect cancer patients' mental and physical well-being 141-143. At the same time, cancer physicians and the medical staff have a tough job balancing the risk of admitting cancer patients to the hospital with the possibility of this vulnerable population getting COVID-19. The shortage of medical staff and the deprivation of resources due to their increased need for COVID-19 care further add to challenges cancer care professionals have so far faced during the pandemic. Therefore, many health care agencies have altered treatment guidelines on cancer care 144, 145. The standard strategy recommended is the prioritization approach, where the risk of hospital admission and the benefit of therapeutic intervention are analyzed and balanced 146, 147. The factors that could influence risk/benefit analysis include the patient's health, cancer status, severity, risk factors associated with severe COVID-19 illness, and patient's preferences. In addition, certain types of cancer treatments are highly immunosuppressive 43, 148. Therefore, decisions regarding delay or initiation of therapies need to be carefully evaluated. As per the American Society of Clinical Oncology (ASCO) guidelines, when deciding to reschedule or modify systemic cancer therapy, one should consider an individualized risk/benefit assessment that includes the overall goals of the treatment, cancer progression risks, patient tolerance of treatment, and the patient's general medical condition 149.

Another challenge physicians will contend with are ethical considerations and the rationing of healthcare by considering patients likely to benefit the most 150. For example, in crowded hospitals, if a COVID-19 patient with a late-stage disease or worsening health conditions, like heart or lung dysfunction, requires life-saving equipment like a ventilator, their chances of survival are low. The concept of allocating scare resources during the pandemic is not new. In World War II, most of the penicillin production in the U.S. was being used for soldiers and there was not enough to meet the need of everyone 151. There have been reports of implementing ethical frameworks for rationing scarce health resources in some countries as the number of hospitalized COVID-19 patients has outstripped the supply of crucial resources like ventilators 152, 153. To cope with these kinds of ethical dilemmas, where physicians have to choose between patients and consider their safety in providing care to infected persons, discussion among the cancer care team, medical ethicists, and palliative care team members is critical.

Furthermore, it should be recommended to increase the use of telemedicine in cancer care. Telemedicine implies the use of a variety of communication means to support clinical care 154. For instance, cancer patients and oncologists can share and discuss patient-related data through mobile phones or computer networks using the internet. Using telemedicine, an oncologist can guide or give medical advice to cancer survivors at home, minimizing their visits to the hospital during this pandemic. This can improve access and care, decrease costs, and protect cancer survivors from unnecessary infections. Moreover, the multidisciplinary management tumor board meetings, that include physicians across multiple specialists, should be the main body in making treatment-related decisions in the COVID-19 era 155. These boards should consider working together through collaborations across the globe to improve patient care and overcome challenges faced during a pandemic.

**European Society for Medical Oncology (ESMO) guidelines:** ESMO issued cancer patient management guidelines during the COVID-19 pandemic 144. As per ESMO, physicians must discuss cancer treatment initiation decisions or continuation with patients and determine if they are fit to be treated and willing to do so after a proper risk/benefit explanation. Hospitals should consider a prioritization approach in delivering cancer care. High priority should be given to patients whose conditions are life-threatening, clinically unstable, and/or the magnitude of benefit qualifies the intervention (e.g., gains in overall survival and/or quality of life). Medium priority patients are not critical, but a delay in intervention beyond six weeks could potentially impact the overall outcome and/or the magnitude of benefit. Low priority patients have stable conditions, and intervention does not change the magnitude of benefit (no gains in overall survival and/or quality of life). In these patients, services can be delayed during the pandemic. Suppose local treatment (surgery or radiation) for early stage cancer is planned. In that case, doctors are advised to explore the “wait and see” approach (which means cancers that are not causing any symptoms or problems are carefully monitored but not treated) or to perform a cost-benefit analysis (comparison of interventions and their consequences) according to the patient's age, comorbidities, and impact of the outcome of the surgical procedure for treatments planned. Also, if available, cancer or palliative intravenous therapies can be temporarily switched to oral medicines 144.

Although such changes in the usual standard of care of such cancer patients is appropriate and necessary, it is highly probable that it will increase morbidity or even mortality in cancer patients.

## 8. Repurposing cancer drugs against COVID-19 treatment

Drug and vaccine development is a slow, complicated, and costly process 156. An alternative approach is to identify existing drugs that can be used for new therapeutic purposes, like for the treatment of COVID-19. This is called drug repurposing or drug repositioning, and around 30% of approved drugs in 2017 are repurposed drugs 157, 158. If it is successful, this approach is very beneficial, especially as there is an immediate need for the COVID-19 therapeutics. Clinical trials of repurposed drugs for COVID-19 treatment are in progress 159-162. There are some similarities between cancer and COVID-19 symptoms, therefore repurposing cancer drugs for COVID-19 treatment might help patients. For example, severe COVID-19 cases are associated with developing a condition similar to acute respiratory distress syndrome 163. This is due to the excessive release of pro-inflammatory cytokines released by immune cells due to lung injury caused by SARS-CoV-2 164. This cytokine storm is one of the common causes of mortality associated with the COVID-19 pandemic 165. It causes widespread tissue damage and fibrosis that occurs during the healing process and can result in persistent organ dysfunction. Some clinically approved anti-cancer drugs inhibit inflammation, and hence could help people with severe cases of COVID-19. One example is ruxolitinib, a Janus kinase inhibitor used for the treatment of myelofibrosis, a cancer of bone marrow that disrupts the normal production of blood cells 166. Ruxolitinib inhibits the activation of a broad range of pro-inflammatory cytokines and growth factors, including TNF-α, IFNγ, IL-1, IL-6, IL-8, IL-12, TGFβ, VEGF, FGF, PDGF, GM-CSF, and G-CSF 167. Ruxolitinib was given to severe COVID-19 patients (n=14) who were at high risk of inflammation and who were deteriorating while receiving standard of care treatment. Patients received ruxolitinib over a median of 9 days with a median cumulative dose of 135 mg. Ruxolitinib treated patients responded well to the treatment and had significantly reduced inflammation and related parameters, including a decrease in CRP levels, IL-6, ferritin, and lymphopenia. Out of 14 patients, ten were recovered, and the four that did not respond well had preexisting comorbidities 168.

Another anti-cancer drug, acalabrutinib, which inhibits Bruton tyrosine kinase (BTK) signaling, proved to be effective in treating severe COVID-19 cases 169. Acalabrutinib is used to treat a type of non-Hodgkin lymphoma known as mantle cell lymphoma 170. In response to certain viral pathogens like SARS-COV-2, Toll-like receptors present on the surface of macrophages activates BTK to mediate the innate immune response 169, 171. BTK activates nuclear factor-kB (NF-kB) signaling, which triggers the production of multiple pro-inflammatory cytokines and chemokines 172. BTK inhibitors prove effective in reducing excessive inflammation and hence could be a therapeutic strategy to treat severe COVID-19 cases. Acalabrutinib treatment in severe COVID-19 patients (n=19) that are on supplemental oxygen (n=11) or mechanical ventilation (n=8) significantly improved oxygenation and lower intubation. Before treatment, 18 of 19 (95%) patients had significantly elevated levels of CRP (n=15: CRP≥10 mg/dl, n=4: CRP=3-10 mg/dl), serum ferritin (n=16, ferritin≥500 ng/ml, n=3: ferritin <500 ng/ml), fibrinogen (>400 mg/dl, n=10), D-dimer (>0.5 μg/ml: n=15), IL-6 (>15 pg/ml) and absolute lymphocyte count (n=15: ≤1000 cells/μl and n=3: >1000 cells/μl). Acalabrutinib was given 100 mg orally per enteric feeding tube twice daily for 10 days to patents on supplemental oxygen and 14 days to patients on mechanical ventilation. In the supplemental oxygen cohort (n=11), 8 patients (75%) no longer needed extra oxygen and had been discharged and the other 3 patients had a decreased oxygen requirement. In the cohort of patients receiving mechanical ventilation, 4 patients (50%) were successfully extubated and two of them were discharged. Three patients remained intubated with oscillating oxygen requirements and one died after the withdrawal of support. In both cohorts, those who responded well had CRP and IL-6 levels that either decreased or returned to normal and absolute lymphocyte counts were significantly improved or back to normal. *Ex vivo* analysis of whole blood samples of these patients (n=3) showed an increase in phosphorylation of BTK (Y223) in CD14+ monocytes compared to healthy individuals (n=5). Additionally, the percentage of IL-6+CD14+ monocytes were also increased in patients with severe COVID-19 (n=4) compared to healthy individuals (n=5), suggesting activated BTK signaling in patients' monocytes due to SARS-COV-2 infection 169.

Interferon-alpha-2b, an anti-proliferative drug that also has antiviral properties, showed a positive effect on the recovery of COVID-19 patients 173. Interferon-alpha-2b is used for the treatment of AIDS-related Kaposi sarcoma, hairy cell leukemia, and melanoma 174. Interferon-alpha-2b binds with type 1 interferon receptors, including IFNAR1 and IFNAR2c, and induces their dimerization. The dimerized receptors activate two Janus kinase (JAK) tyrosine receptors, Jak1 and Tyk2, that trans-phosphorylate themselves and the receptors. Phosphorylated receptors bind and activate signal transducers and activators of transcription (STAT) 1 and 2. Activated STATs dimerize and activate multiple immunomodulatory and antiviral proteins 175. Interferon alpha-2b treatment reduces viral load and elevated levels of IL-6 and CRP in COVID-19 patients. Treatment of patients (n=77) with interferon-alpha-2b alone or in combination with the broad-spectrum antiviral drug arbidol reduces viral clearance in 20.3 and 21.1 mean days, respectively, compared to 27.9 mean days in the arbidol treatment alone group. The circulating IL-6 and CRP levels in the arbidol treatment group were higher than the patients treated with IFN alone or in combination with arbidol. This suggests that interferon-alpha-2b could be beneficial in lowering the viral load and related symptoms in COVID-19 patients. It is noted that the patients treated here did not have respiratory distress that required prolonged oxygen supplementation or intubation, suggesting moderate cases of COVID-19 175. Furthermore, interferon-alpha-2b in combination with arbidol accelerates pneumonia absorption and significantly improved chest CT scans in patients with mild COVID-19 176. Interferon-alpha-2b in combination with rintatolimod are in clinical trials for the treatment of mild or moderate COVID-19 in cancer patients (TI: NCT04379518).

Another antiviral drug, remdesivir, a nucleotide analog prodrug that inhibits viral RNA polymerases, has shown promising results against COVID-19 in many studies 177-181. Remdesivir is not an anti-cancer drug and was also not approved by the FDA to treat or prevent any disease before the COVID-19 crisis. It is important to mention remdesivir here because the U.S. FDA gave emergency use authorization for remdesivir for COVID-19 treatment to all hospitalized patients 182. The use of remdesivir in patients with COVID-19 significantly improves their clinical outcome 177. Of the 53 patients who had received remdesivir, 68% showed an improvement in the category of oxygen support over a median follow-up of 18 days. All patients (n=12) who received low-flow supplemental oxygen or non-invasive oxygen support showed improvement. Moreover, by the most recent follow-up, 47% of patients had been discharged (24% were receiving invasive ventilation, and 89% were receiving non-invasive oxygen support). Thirteen percent of patients had died after completing the treatment, and mortality was high in aged patients (>70 years) or those on invasive ventilation 177. Beigel et al. reported similar findings in their clinical trial of intravenous remdesivir in adults (n=538) hospitalized with COVID-19 181. Remdesivir treatment significantly shortened the recovery time in COVID-19 patients compared to placebo control. The median recovery time was 11 days and mortality was 7% by 14 days after enrollment in the patients treated with remdesivir compared to placebo control 181. Furthermore, a clinical trial by Gilead Sciences showed that remdesivir treated COVID-19 patients had improved clinical recovery and a 62% reduction in the risk of mortality compared to standard of care 178. Many clinical trials are underway, seeking the effects of remdesivir in the treatment of COVID-19 183, 184.

In conclusion, the present clinical management includes prevention of infection and providing supportive care, such as oxygen supplementation, mechanical ventilatory support, and some investigational drugs. Anti-cancer drugs can be used to treat inflammation, immune dysfunction, and viral multiplication and they are safe and effective. Therefore, repurposing of cancer drugs could be a smart choice to treat the COVID-19 pandemic, which is spreading fast and creating havoc all over the world. Table 3 shows the anti-cancer drugs that are in investigational use for the treatment of COVID-19.

## Conclusions and Future Perspectives

The COVID-19 pandemic is a threat to human health worldwide. As stated, there are no approved therapies for the treatment of COVID-19. Therefore, extensive research would help in developing therapeutic molecules to combat this pandemic. Still, it is unknown why certain people respond differently to SARS-CoV-2 infection. Compared to the healthy population, people with existing comorbidities like cancer are more vulnerable to severe outcomes of COVID-19. Therefore, cancer patients should be extra cautious, and hospitals should have better management plans to mitigate the adverse effects of the COVID-19 pandemic on vulnerable cancer populations. The postponement of chemotherapy or surgery, intensive treatment, more personal protection, telecommunication, and a separate treatment strategy for the treatment of COVID-19 patients with cancer should be recommended. Since, SARS-CoV-2 is an emerging infection to humans around the world, no matter how advanced the medical fields are, many more cross-species infections are expected in the future. Therefore, it is also essential to surveil for other viruses to enhance our preparedness for future outbreaks.

## Figures and Tables

**Table 1:** Effect of cancer treatment on COVID-19 severity in cancer patients

References	Type of treatment	% Outcomes (M=mortality and SS=severe symptoms) *NS: non-significant*
**19**	Immunotherapy	M: 33.33 vs. 5 (no-cancer) and SS: 66.8 vs. 16 (no-cancer)
	Surgery	M: 25 vs. 5 (no-cancer), and SS: 62.5 vs. 16 (no-cancer)
	Chemotherapy	M: 12 vs. 5 (no-cancer), NS and SS: 41 vs. 16 (no-cancer)
	Radiotherapy	M: 8 vs. 5 (no-cancer), NS and SS: 23 vs. 16 (no-cancer) NS
**99**	Immunotherapy or target therapy	SS: 81, non-SS: 19
	Surgery	SS: 40, non-SS: 60
	Chemotherapy or radiotherapy	SS: 34, non-SS: 66
**135**	Immunotherapy	SS in lung cancer: 58 vs. 35 (non-immunotherapy treatment) SS in other solid cancers: 26 vs. 15 (non-immunotherapy treatment)
	Surgery	*NS* difference in groups (treated vs. not treated with surgery)
	Chemotherapy	*NS* difference in groups (treated vs. not treated with chemotherapy)
**56**	Immunotherapy	M: 33 vs. 27 (no-treatment), *NS*
	TKI therapy	M: 29 vs. 27 (no-treatment), *NS*
	Chemotherapy	M: 48 vs. 27(no-treatment)
**194**		Treatments within 4 weeks before symptom onset
	Immunotherapy	M: 1 vs. 6 (live), *NS*
	Surgery	M: 3 vs. 0 (live), *NS*
	Chemotherapy	M: 11 vs. 44 (live)
	Radiotherapy	M: 4 vs. 9 (live) *NS*
	Target therapy	M: 4 vs. 18 (live)
**195**	Chemotherapy	Patients with haematological malignancies having recent chemotherapy treatment are at higher risk of death during COVID-19-associated hospital admission (OR 2·09, 95% CI 1·09-4·08; p=0·028)
**47**	Non-cytotoxic therapies: Immunotherapy, radiotherapy, target therapy and endocrine therapy	Treated vs not treated within 4 weeks of COVID-19 diagnosis M: 11 vs. 14, *NS* SS: 24 vs. 28, *NS*
	Surgery:	Treated vs not treated within 4 weeks of COVID-19 diagnosis M: 19 vs. 13, *NS* and SS: 38 vs. 26, *NS*
	Chemotherapy	Treated vs not treated within 4 weeks of COVID-19 diagnosis M: 14 vs. 14, *NS* and SS: 22 vs. 28, *NS*
**196**	Immunotherapy, Surgery, Chemotherapy, Radiotherapy, Hormone therapy and Target therapy	No significant effect on mortality for patients who had the therapies in the past 4 weeks compared to non-treated patients
**55**	Immunotherapy (anti-PD-1), Chemotherapy and TKI therapy	No significant difference in M and SS vs. non-treated patients with respective therapy

**Table 2:** Treatment strategies used for COVID-19 treatment in cancer patients

References	Cancer type	Treatment strategies
**45**	Lung cancer	HCQ + azithromycin (n=8), HCQ + lopinavir/ritonavir (n=2), HCQ+ azithromycin + lopinavir/ritonavir (n=1), HCQ (n=1)
**85**	Breast cancer	Levofloxacin, piperacillin plus tazobactam combined with the antiviral combination of darunavir/cobicistat. To this combination, HCQ was added (n=1)
**197**	Multiple cancers	Remdesivir (n=1), Azithromycin (n=138), Tocilizumab (n=6), HCQ (n=150), Convalescent plasma (n=9), Systemic corticosteroids (n=117)
**47**	Solid and hematological malignancies	HCQ (n=89), Azithromycin (n=93), HCQ+ azithromycin (n=181)
**198**	Chronic lymphocytic leukemia	HCQ (n=108), Remdesivir (n=14), Lopinavir/Ritonavir (n=34, Tocilizumab (n=43), Intravenous immunoglobulin, (n=13.8), Corticosteroids (n=95), Azithromycin (n=53), Convalescent Plasma (n=10)
**199**	Pediatric cancers	Broad-spectrum antibiotics (n=5), HCQ (n=5), Lopinavir/Ritonavir (n=1), Oxygen support
**200**	Multiple cancers	Antibiotics (n=138), HCQ (n=107), Lopinavir/ritonavir (n=84), Corticosteroids (n=41), Corticosteroids (n=15), Tocilizumab (n=9)
**201**	Prostate cancer	Study included patients receiving ADT for cancer treatment (n=22) vs non-ADT group (n=36). ADT use was defined as hormones that lower the level of testosterone like Gonadotropin-releasing hormone (GnRH) analog or antagonist administered within three months and/or documented testosterone concentrations < 50 ng/dl within six months of COVID-19 diagnosis
**TI:** **NCT04446429**	Prostate cancer and benign prostatic hyperplasia	It is a clinical trial to explore the protective role of anti-androgen drugs dutasteride and proxalutamide in combination with ivermectin and azithromycin against COVID-19

n=number of patients, TI: Trial identifier (ClinicalTrials.gov Identifier)

**Table 3:** Repurposed cancer drugs in clinical trials for COVID-19 treatment

Cancer drugs	Approved for cancer type	Mechanism of action	Clinical trials for COVID-19
Duvelisib	Chronic lymphocytic leukemia and small lymphocytic lymphoma	Inhibitor of phosphoinositide 3-kinase (PI3K) delta and gamma isoforms 202	Dose: 25 mg twice daily for 10 days. TI: NCT04372602
Isotretinoin (13-cis-retinoic acid)	Neuroblastoma	Exact mechanism unknown. Induces apoptosis and cell cycle arrest 203-208	Dose: 0.5 mg/kg/day in 2 divided doses orally for one month. TI: NCT04361422
Decitabine	Myelodysplastic syndrome	DNA hypomethylating agent. Inhibits DNA methyltransferases 209	Dose: 10mg/m^2^ body surface iv for 5 days. TI: NCT04482621
Dexamethasone	Used with other drugs to treat leukemia and lymphoma	Anti-inflammatory and immunosuppressive. Inhibits the expression of inflammatory mediators 210	Dose: 20 mg/iv/daily for 5 days, followed by 10 mg/iv/daily for 4 days. TI: NCT04325061
Etoposide	Multiple cancers including testicular, lung, lymphoma, leukemia, neuroblastoma, and ovarian cancer	Form stable complex with DNA and topoisomerase II enzyme. This prevents repair by topoisomerase II and induces double stranded DNA breaks 211	Dose: 150mg/m^2^ body surface iv once daily on days 1 and 4. If patient benefits but have cytokine storm symptoms treatment continue on day 8, 11, 18 and 25. TI: NCT04394416
Imatinib mesylate	Acute lymphoblastic leukemia, chronic myelogenous leukemia**,** gastrointestinal stromal tumor, and myelodysplastic syndrome	Protein tyrosine kinase inhibitor. Inhibits bcr-abl tyrosine kinase 212	Dose: 400 mg daily, oral for 14 days. TI: NCT04394416
Interleukin 2 (IL-2)	Metastatic renal cell carcinoma and melanoma	Activates CD8+ T and NK cells 213	Dose: Subcutaneous injections, once-daily administration for 10 days. TI: NCT04357444
Nintedanib	Non-small cell lung cancer	Binds with ATP binding pocket of fibroblast growth factor, platelet-derived growth factor and vascular endothelial growth factor receptors resulting in blockage of the autophosphorylation of these receptors and the downstream signaling cascades	Dose: 150 mg capsule, twice a day, about 12 hours apart for 8 weeks. TI: NCT04338802
Lenalidomide	Myelodysplastic syndrome, multiple myeloma, and mantle cell lymphoma	Inhibits cell proliferation, angiogenesis and promotes immune response. Inhibits cyclooxygenease-2 (COX-2). Promotes the ubiquitination of transcription factors IKZF1 and IKZF3 214, 215	Dose: 5mg capsule orally daily, on days 1,3 and 5 together with a prophylactic dose of low molecular weight heparin. TI: NCT04361643
Prednisone	Acute lymphoblastic leukemia, Chronic lymphocytic leukemia Hodgkin and Non-Hodgkin lymphoma	Inhibits NF-kB and other inflammatory transcription factors 216	Dose: Orally, 0.75 mg/kg/day for 5 days, then 20 mg/day for 5 more days. TI: NCT04344288
Tamoxifen	Breast cancer	Binds to estrogen receptor and blocks its proliferative actions 217	Dose: 20mg orally twice daily for 14 days. TI: NCT04389580
Zanubrutinib	Mantle cell lymphoma	Binds and inhibits Bruton's tyrosine kinase (BTK) activity 218	Dose: 320 mg (4 x 80 mg) capsules orally once daily up to 28 days. TI: NCT04382586
Methotrexate	Multiple cancers including breast, advanced head and neck, lung, stomach and blood cancers	Inhibits enzymes involved in nucleotide synthesis including dihydropholate reductase which results in the deficiency of nucleotide pools to be used in nucleic acid synthesis 219	Dose given in phases: Phase 1: 20mg/week for 4 weeks. Phase 2: 30mg/week for 4 weeks. Phase 3: 40mg/week for 4 weeks. TI: NCT04352465
Proxalutamide	Prostate cancer	Androgen receptor antagonist 220	Dose: 200 mg with standard care or with 200 μg/kg ivermectin and 500 mg azithromycin once a day TI: NCT04446429
Dutasteride	Benign prostatic hyperplasia	It inhibits 5α-reductase enzymes that convert testosterone into dihydrotesterone (DHT) and reduces its levels 221	Dose: 0.5 mg with standard care or with 200 μg/kg ivermectin and 500mg azithromycin once a day. TI: NCT04446429
Remdesivir	Not an anti-cancer drug	Nucleotide analog prodrug, which inhibits viral RNA polymerases 222	Dose: 100-200 mg with or without standard care (TI: NCT04292899 and NCT04292730) or other drugs including tocilizumab (TI: NCT04409262), HCQ (TI: NCT04345419), Baricitinib (TI: NCT04401579)

TI: Trial identifier (ClinicalTrials.gov Identifier)
